# Medullospheres from DAOY, UW228 and ONS-76 Cells: Increased Stem Cell Population and Proteomic Modifications

**DOI:** 10.1371/journal.pone.0063748

**Published:** 2013-05-24

**Authors:** Cristina Zanini, Elisabetta Ercole, Giorgia Mandili, Roberta Salaroli, Alice Poli, Cristiano Renna, Valentina Papa, Giovanna Cenacchi, Marco Forni

**Affiliations:** 1 EuroClone S.p.A Research Laboratory, Molecular Biotechnology Centre (MBC), University of Turin, Turin, Italy; 2 Department of Experimental Medicine and Oncology (CERMS), University of Turin, Turin, Italy; 3 Department of Biomedical and Neuromotor Sciences, “Alma Mater Studiorum” University of Bologna, Bologna, Italy; 4 BioDigitalValley S.r.l., Pont Saint Martin (AO), Italy; University of Medicine and Dentistry of New Jersey, United States of America

## Abstract

**Background:**

Medulloblastoma (MB) is an aggressive pediatric tumor of the Central Nervous System (CNS) usually treated according to a refined risk stratification. The study of cancer stem cells (CSC) in MB is a promising approach aimed at finding new treatment strategies.

**Methodology/Principal Findings:**

The CSC compartment was studied in three characterized MB cell lines (DAOY, UW228 and ONS-76) grown in standard adhesion as well as being grown as spheres, which enables expansion of the CSC population. MB cell lines, grown in adherence and as spheres, were subjected to morphologic analysis at the light and electron microscopic level, as well as cytofluorimetric determinations. Medullospheres (MBS) were shown to express increasingly immature features, along with the stem cells markers: CD133, Nestin and β-catenin. Proteomic analysis highlighted the differences between MB cell lines, demonstrating a unique protein profile for each cell line, and minor differences when grown as spheres. In MBS, MALDI-TOF also identified some proteins, that have been linked to tumor progression and resistance, such as Nucleophosmin (NPM). In addition, immunocytochemistry detected Sox-2 as a stemness marker of MBS, as well as confirming high NPM expression.

**Conclusions/Significance:**

Culture conditioning based on low attachment flasks and specialized medium may provide new data on the staminal compartment of CNS tumors, although a proteomic profile of CSC is still elusive for MB.

## Introduction

Medulloblastoma (MB) is an aggressive pediatric tumor of the cerebellum with “embryonal” features and early leptomeningeal spreading.

A dramatic increase in crude survival has been associated with relevant toxicity as a result of chemotherapy and/or radiation therapy effects on the developing brain.

A wealth of new data, from the new pathological classification [1] to genetic studies based on gene expression and Comparative Genomic Hybridization [2], as well as Proteomics [3], has permitted the identification of molecular subgroups with different gene expression profiles and protein expression. A therapeutic approach based on the risk stratification of patients may ensure a better quality of life to children that are treated in order to avoid over-treatment.

A better understanding of the role of Cancer Stem Cells (CSC), (recently also referred as brain tumor-initiating cells) may be of peculiar interest in MB, a tumor with relevant molecular heterogeneity [4]. A validated method to study CSC *in vitro* is through cell culture [5], [6] by creating a neurosphere assay (NSA). DAOY, UW228 and ONS-76 are well-known MB cell lines, and are considered to be representative of a primary MB [7], [8]. In this study we used these cell lines as a model for evaluating progression and malignancy of MB and to investigate modifications induced by sphere formation. It is worth noting that ONS-76 has been described as a more immature cell line with a primitive profile, able to differentiate towards a neuronal phenotype [9]. Conversely, UW228 are characteristically less invasive, with a slower rate of cell division [10]. As already reported, CSC showed high expression of markers such as CD133, CD44, Nanog and Oct4 and are considered signs of stemness also in MB [11]. Nestin and SOX-2 play a role in neurogenesis and are considered to be markers of neural stem cells in brain development [12].

Proteomic analysis of MB subtypes may be of interest not only to refine stratification of patients into risk categories but also to give new insights into the elusive existence of CSC.

With the present study we report our experience in culturing tumor cells derived from MB in a serum-free culture medium resulting in the formation of spheres. We applied proteomic techniques to evaluate variations in protein expression, and the possible relation to relevant modifications in biological behavior, such as aggressiveness and therapy resistance. Mass spectrometry analysis did not confirm a unique proteomic profile for CSC generated from the three cells line of MB. Only a few protein modifications were found in MBS without any strong evidence of enrichment in CSC.

## Results

### Morphology MBS Cell Lines

**Figure 1:**
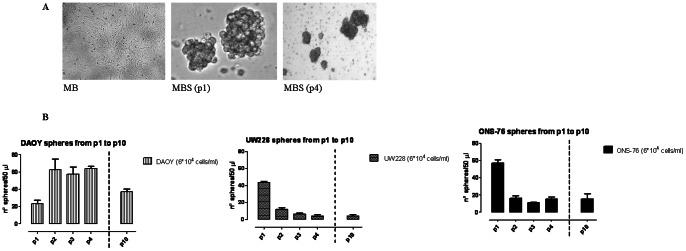
Morphology and medullosphere count derived from adherent MB tumor cells. (**A**) Representative morphology of adherent cells and medullospheres in P1 and P4. (**B**) MBS count obtained with MB cell lines during different passages (P1–P10). Measurements were done in triplicate and data are presented as mean ± SD.

MBS were prepared from established MB cell lines and expanded in serum-free medium. Figure 1 A shows a representative morphology feature of adherent and sphere cell lines.

ONS-76 formed large spheres (142.77 µm±74.07) compared to DAOY (45.70±12.65) and UW228 (42.03±7.81). Furthermore, DAOY spheres were more fragile and more susceptible to destruction during manipulations.

Since the conditions of culture for sphere formation are the same the different size of spheres is related mainly to intrinsic proprieties of each cell line when grown as spheres. Cells were successfully amplified in medullospheres during more than 10 passages with variable amounts of spheres obtained at each passage depending on cell line. All MB cell lines continuously formed MBS with successive passages after. DAOY showed a number of spheres that increased in the first four passages but decreased during subsequent passages. Conversely, UW228 and ONS-76 showed a different trend, with the number of spheres decreasing from the second passage onwards (Fig. 1B).

### Transmission Electron Microscopy

**Figure 2:**
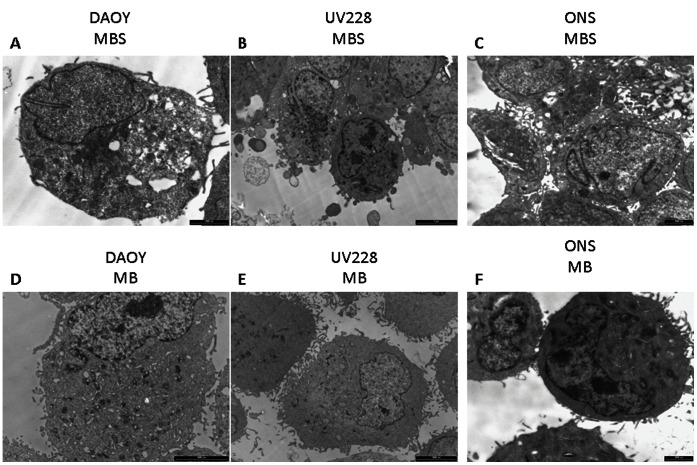
Transmission electron microscopy. MBS from DAOY (**A**), UW228 (**B**) and ONS-76 (**C**) show a uniform stem-like aspect characterized by a high N:C ratio; nucleus is irregularly shaped and cytoplasm appears sparsely organized with unspecific electron-dense organelles (**B**) and vacuoles (**A**). MB cell lines feature a low N:C ratio (**D, E**): nuclei exhibit an irregular profile and are peripherally located; the cytoplasm is populated by many organelles, such as RER, Golgi apparatus and mitochondria. On the contrary, ONS MB seem to maintain stemness appearance (**F**) as seen in the high N:C ratio with a kidney-shaped nucleus rimmed by a thin layer of cytoplasm.

DAOY MBS generally show a stem-like appearance: featuring a high nucleus: cytoplasm, (N:C) ratio. The nucleus is irregularly shaped and the cytoplasm is sparsely organized. Only focal RER profiles arranged in a concentric way are visible. DAOY MB cell lines show a more represented cytoplasm with a lower N:C ratio. In the cytoplasm there are numerous RER profiles, Golgi apparatus and focal mitochondria aggregates. (Fig. 2A,D).

ONS-76 MBS confirmed many of the aspects described for DAOY MBS, such as the stem-like appearance characterized by a high N:C ratio. The ONS-76 MB cell line also shows a similar sub-microsopic aspect featuring very little cytoplasm largely occupied by a prominent nucleus with a kidney-shaped appearance (Figure 2 C,F).

UV228 MBS cells seem to recapitulate the ultra-structural aspects already described for DAOY MBS. Sometimes a rudimentary-organized RER is visible in cells with a high N:C. The UW228 MB cell line shows a peripheral kidney-shaped nucleus (Figure 2 C,E) and a well-represented cytoplasm organized with focal intermediate filament aggregates. Only a few stem-like cells are recognizable.

### Cytofluorimetric Analysis of MB and MBS Cell Lines

MB cell lines show a considerably undifferentiated phenotype. They are plastic-adherent when maintained in expansion conditions and express certain progenitor markers such as CD44, Nanog and Oct4 at varying levels. Table 1 shows all results of marker expression for all cell lines, both in adherent conditions and in the correspondent spheres.

**Table 1:** Phenotypic analysis of stemness marker expression in medulloblastoma cell lines and in correspondent spheres.

*Progenitor markers*	*DAOY*				*UW228*				*ONS-76*			
	*MB*		*MBS*		*MB*		*MBS*		*MB*		*MBS*	
CD133	**5.92**	**±4.37**	**92.14**	**±9.87**	**1.03**	**±0.85**	**49.35**	**±7.23**	**54.53**	**±1.45**	**90.57**	**±1.44**
CD44	96.19	±5.30	97.41	±3.24	99.01	±1.36	95.21	±5.35	91.01	±1.23	98.81	±0.45
Oct4	94.06	±4.65	90.78	±0.78	**62.30**	**±6.87**	**95.45**	**±2.37**	0.13	±0.13	0.74	±0.64
Nanog	93.88	±5.22	90.95	±0.72	94.27	±4.78	93.33	±3.45	0.59	±0.91	1.39	±1.12
β-catenin	**1.09**	**±0.21**	**83.85**	**±1.97**	**64.85**	**±7.70**	**93.99**	**±4.53**	**11.36**	**±0.93**	**81.54**	**±2.04**
Nestin	**8.83**	**±1.25**	**82.43**	**±9.56**	**1.22**	**±0.41**	**29.66**	**±4.67**	**50.71**	**±5.67**	**89.79**	**±3.25**

The table represents mean standard deviation of three independent experiments. Numbers in bold indicate a statistically significant difference of spheres with reference to the control adhesion conditions (*p<0,01).

DAOY and UW228 adherent cell lines do not express neuronal stemness markers such as Nestin, or the very early progenitor markers such as CD133. In adherent conditions, ONS-76 cell lines express the same neuronal markers at high levels (Nestin: 50.7±5.7 and CD133∶54.5±1.4). Β-catenin, a protein involved in the WNT signaling pathway was expressed in variable levels according to cell type; in DAOY (β-catenin: 1.09±0.21), UW228 (β-catenin: 64.8±7.7) and ONS-76 (β-catenin: 11.4±0.9).

**Figure 3:**
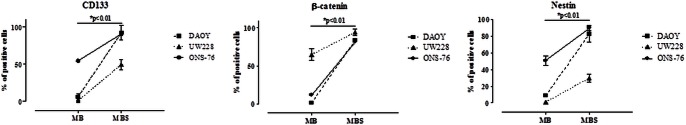
Analysis of MB and MDB expression by Flow cytometry. FACS analyses shows expression of progenitor markers, CD133 (**A**) beta- catenin (**B**) and nestin (**C**)**,** evaluated in both MB and in MBS for all cell lines by flow cytometry. Graphs represent pooled data from three independent determinations. Standard deviations and Student’s T-test were calculate to compare MB and MBS. Probability values less than 0.01 were considered significant.

MBS were tested for the same protein expression, and DAOY and UW228 cell lines showed a general and evident enrichment in stem cells, featuring increased levels of Nestin, β-catenin and CD133 in comparison with MB adherent cell lines. Expression is variable but marked, both for DAOY (Nestin: 82.4±9.6; β-catenin: 83.8±2.0 and CD133∶92.1±9.9) and for UW228 (Nestin: 29.7±4.7; β-catenin: 94.0±4.5 and CD133∶49.3±7.2). ONS-76 cells lines were also shown to be positive for the same neuronal stemness markers (Nestin: 89.8±3.2; β-catenin: 81.5±2.0 and CD133∶90.6±1.4). Values of positive cells for neuronal stemness markers in MB and MBS for all three cells lines are represented in Figure 3.

### In vitro Invasion Capability of MB and MBS Cell Lines

**Figure 4:**
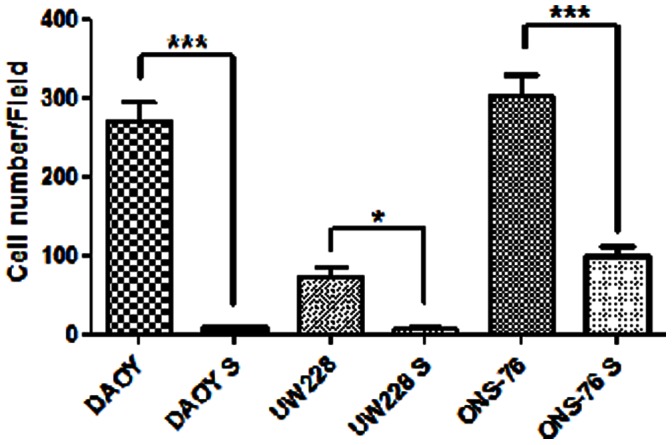
Migration assay. Invasion capability of MB cells (ONS-76, DAOY and UW228) and their corresponding MBS (ONS-76 S, DAOY S and UW228 S) at 24h from experimental set up using a transwell (Matrigel) migration assay. The results are representative of two different experiments. Bar charts represent the mean of migrating cells evaluated in two independent experiments. *P<0,05 ***P<0,0001 (unpaired, two tailed t-test).

Migration capability was different for our MB adherent cell lines: ONS-76 migrated more than DAOY and DAOY migrated much more than UW228. On the other hand, all MBS cell lines showed a statistically significant decrease in invasion capability respect to adherent cell lines (Fig. 4).

### Proteomics

To identify proteins involved in spheres formation three independent 2-DE experiments on cellular lysate from DAOY, UW228 and ONS-76 in adherent conditions versus spheres at first passage (P1) were performed.

**Figure 5:**
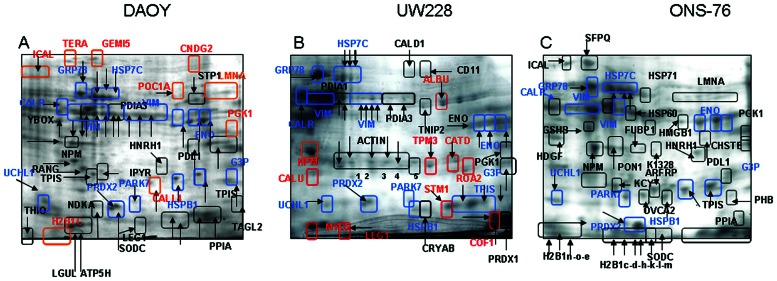
Proteomic analysis. Representative image from three independent experiments of Coomassie-stained 2-DE patterns of DAOY(**A**), UW228 (**B**) and ONS-76 (**C**) cell lines. Proteins were separated using 7-cm, pH-3-10 NL strips followed by SDSPAGE on 10%, 7×10-cm gels. Proteins showing differential expression were indicated with different colors: in *blue* –proteins identified in all cell lines and listed in Table 2; in *black*-proteins identified in adherent cells and spheres of each cell lines and in *red*-proteins only identified in spheres. Corresponding identifications are reported in Table 3, 4 and 5.

Figure 5 shows the 2-DE pattern from MBS (DAOY Fig. 5A, UW228 Fig. 5B and ONS-76 Fig. 5C) in the pH range 3–10 NL after Blue Coomassie staining. 2-DE maps from controls and spheres show a very similar proteomic pattern with differences in distribution and spot intensities of just a few proteins. The 50 most intense spot per gel were cut and analyzed by mass spectrometry.

After MALDI-TOF analysis and a database search, 300 spots were characterized. Many spots were isoforms of the same protein, while others were not identified with an acceptable score. From these, 77 unique proteins were well identified: 11 proteins CALR, ENOA, G3P, GRP78, HSP7C, HSPB1, PARK7, PRDX2, TPIS, UCHL1, VIME (indicated in blue in Figure 4) were differentially expressed in all DAOY, UW228 and ONS-76 adherent and sphere cell lines (Table 2). These proteins are probably most abundant in the of MB cell lines as we have already identified their presence in a set of MB histotypes, as previously described [3].

**Table 2:** Proteins identified by MALDI-TOF in all (DAOY, UW228, ONS-76) Adherent and Sphere cell lines.

*Abb* a	*AC* b	*name*	*MW* c	*p.I.* d	*MVM* e	*C%* f	*MS* g	*CC* h
CALR_HUMAN	P27797	Calreticulin	48283	4,29	8	30	81	Adh/Sp
ENOA_HUMAN	Q6GMP2	Alpha-enolase	47481	7,01	9	40	48	Adh/Sp
G3P_HUMAN	P04406	Glyceraldehyde-3-phosphate dehydrogenase	36201	8,57	7	28	74	Adh/Sp
GRP78_HUMAN	P11021	78 kDa glucose-regulated protein	72402	5,07	11	22	104	Adh/Sp
HSP7C_HUMAN	P11142	Heat shock cognate 71 kDa protein	71082	5,37	10	16	76	Adh/Sp
HSPB1_HUMAN	Q9UC36	Heat shock protein beta-1	22826	5,98	8	34	132	Adh/Sp
PARK7_HUMAN	Q99497	Protein DJ-1	20050	6,33	16	72	179	Adh/Sp
PRDX2_HUMAN	P32119	Peroxiredoxin-2	22049	5,66	8	41	124	Adh/Sp
TPIS_HUMAN	P60174	Triosephosphate isomerase	31057	5,65	12	41	158	Adh/Sp
UCHL1_HUMAN	P09936	Ubiquitin carboxyl-terminal hydrolase isozyme L1	25151	5,33	11	65	174	Adh/Sp
VIME_HUMAN	Q8N850	Vimentin	53676	5,06	13	37	128	Adh/Sp

a Abb, abbreviation.

b AC, accession number.

c MW, molecular weight.

d pI, isoelectric point.

e NVM, number of matched mass values on number of total mass values searched.

f C%, the sequence coverage, which is calculated as the percentage of identified sequence to the complete sequence of the matched protein.

g MS, mascot score.

h CC, culture condition.

### Proteins in Medullospheres

DAOY and UW228 modified their proteomic profile from adherent cells to spheres with nine new proteins identified in DAOY MBS (Table 3) and ten in UW228 MBS (Table 4 and indicated in red in Figure 5). Apparently no new proteins were present in ONS-76 cell lines after sphere formation (Table 5) but only differential proteins were expressed. Interestingly, nucleophosmin (NPM) [13] is absent in adherent UW228 cells, but is found in UW228 generated spheres. Expression of NPM is however detected in DAOY and ONS-76 cells with an increased expression in generated spheres. NPM is a major nucleolar phosphoprotein which is more abundant in selected cancer cells than in normal cells, as well as being a marker of drug-resistances in tumor cells. We confirmed NPM expression by immunohistochemistry as a potential marker of biological aggressiveness in MB. From other proteins identified in spheres, Cofilin 1 and Stathmin were also expressed in UW228 spheres. In one of our previous studies [3], we found both these proteins in Desmoplatic and Anaplastic MDB with an increase of stathmin expression in post RT- Anaplastic MB [14].

**Table 3:** Proteins identified by MALDI-TOF only in DAOY Adherent and Sphere cell lines.

*Abb* a	*AC* b	*name*	*MW* c	*p.I.* d	*MVM* e	*C%* f	*MS* g	*CC* h
ATP5H_HUMAN	O75947	ATP synthase subunit d, mitochondrial	18537	5,21	5	47	57	Adh/Sp
HNRH1_HUMAN	P31943	Heterogeneous nuclear ribonucleoprotein H	49484	5,89	7	27	61	Adh/Sp
IPYR_HUMAN	Q15181	Inorganic pyrophosphatase	33095	5,54	13	56	182	Adh/Sp
LEG1_HUMAN	P09382	Galectin-1	15048	5,34	9	73	130	Adh/Sp
LGUL_HUMAN	Q04760	Lactoylglutathione lyase	20992	5,12	6	42	67	Adh/Sp
NDKA_HUMAN	P15531	Nucleoside diphosphate kinase A	17309	5,83	10	78	145	Adh/Sp
PDIA3_HUMAN	P30101	Protein disulfide-isomerase A3	57146	5,98	12	32	134	Adh/Sp
PDLI1_HUMAN	O00151	PDZ and LIM domain protein 1	36505	6,56	9	33	88	Adh/Sp
PPIA_HUMAN	Q9UC61	Peptidyl-prolyl cis-trans isomerase A	18229	7,68	9	74	126	Adh/Sp
RANG_HUMAN	P43487	Ran-specific GTPase-activating protein	23467	5,19	9	43	105	Adh/Sp
SODC_HUMAN	Q6NR85	Superoxide dismutase [Cu-Zn]	16154	5,7	5	38	72	Adh/Sp
STIP1_HUMAN	P31948	Stress-induced-phosphoprotein 1	63227	6,4	9	25	80	Adh/Sp
TAGL2_HUMAN	P37802	Transgelin-2	22548	8,41	7	43	84	Adh/Sp
YBOX1_HUMAN	P67809	Nuclease-sensitive element-binding protein 1	35903	9,87	8	28	79	Adh/Sp
THIO_HUMAN	P10599	Thioredoxin	12015	4,82	4	54	60	Adh/Sp
NPM_HUMAN	Q8WTW5	Nucleophosmin	32726	4,64	7	26	52	Adh/Sp
CALL4_HUMAN	Q96GE6	Calmodulin-like protein 4	21983	7,02	5	23	63	Sp
CNDG2_HUMAN	Q86XI2	Condensin-2 complex subunit G2	130960	06∶43	7	20	48	Sp
GEMI5_HUMAN	Q8TEQ6	Gem-associated protein 5	170821	6,17	7	7	56	Sp
ICAL_HUMAN	P20810	Calpastatin	76572	4,97	8	8	43	Sp
H2B1C_HUMAN	P62807	Histone H2B type 1-C/E/F/G/I	13898	10,31	9	43	84	Sp
LMNA_HUMAN	P02545	Prelamin-A/C	74380	6,57	8	15	63	Sp
PGK1_HUMAN	P00558	Phosphoglycerate kinase 1	44985	8,3	10	28	105	Sp
POC1A_HUMAN	Q8NBT0	POC1 centriolar protein homolog A	45008	07∶31	10	22	46	Sp
TERA_HUMAN	P55072	Transitional endoplasmic reticulum ATPase	89950	5,14	7	14	60	Sp

a Abb, abbreviation.

b AC, accession number.

c MW, molecular weight.

d pI, isoelectric point.

e NVM, number of matched mass values on number of total mass values searched.

f C%, the sequence coverage, which is calculated as the percentage of identified sequence to the complete sequence of the matched protein.

g MS, mascot score.

h CC, culture condition.

**Table 4:** Proteins identified by MALDI-TOF only in UW228 Adherent and Sphere cell lines.

*Abb* a	*AC* b	*name*	*MW* c	*p.I.* d	*MVM* e	*C%* f	*MS* g	*CC* h
ACTA_HUMAN	P62736	Actin, aortic smooth muscle	42381	5,23	8	22	88	Adh/Sp
ACTB_HUMAN	Q96HG5	Actin, cytoplasmic 1	42052	5,29	10	21	60	Adh/Sp
ACTC_HUMAN	P68032	Actin, alpha cardiac muscle 1	42334	5,23	8	22	88	Adh/Sp
ACTG_HUMAN	P63261	Actin, cytoplasmic 2	42108	5,31	6	21	58	Adh/Sp
ACTH_HUMAN	P63267	Actin, gamma-enteric smooth muscle	42249	5,31	6	22	88	Adh/Sp
CALD1_HUMAN	Q05682	Caldesmon	93251	5,63	9	14	78	Adh/Sp
CD11A_HUMAN	Q9UP54	Cell division protein kinase 11A	91146	5,34	6	10	56	Adh/Sp
CRYAB_HUMAN	P02511	Alpha-crystallin B chain	20146	6,76	7	59	150	Adh/Sp
PDIA1_HUMAN	P07237	Protein disulfide-isomerase	57480	4,76	10	25	106	Adh/Sp
PDIA3_HUMAN	P30101	Protein disulfide-isomerase A3	57146	5,98	9	18	84	Adh/Sp
PGK1_HUMAN	P00558	Phosphoglycerate kinase 1	44985	8,3	10	37	108	Adh/Sp
PRDX1_HUMAN	Q06830	Peroxiredoxin-1	22324	8,27	8	61	121	Adh/Sp
TNIP2_HUMAN	Q8NFZ5	TNFAIP3-interacting protein 2	49240	6,03	7	15	66	Adh/Sp
TPM3_HUMAN	P06753	Tropomyosin alpha-3 chain	32856	4,68	8	16	74	Sp
ALBU_HUMAN	Q9P157	Serum albumin	71317	5,92	8	14	63	Sp
CALU_HUMAN	O43852	Calumenin precursor	34961	04∶46	15	11	49	Sp
CATD_HUMAN	P07339	Cathepsin D	45037	6,1	11	26	118	Sp
COF1_HUMAN	P23528	Cofilin-1	18371	08∶26	12	22	55	Sp
LEG1_HUMAN	P09382	Galectin-1	15048	5,34	5	34	61	Sp
MYL6_HUMAN	P60660	Myosin light polypeptide 6	17090	4,56	10	50	112	Sp
ROA2_HUMAN	P22626	Heterogeneous nuclear ribonucleoproteins A2/B1	37464	8,97	9	27	91	Sp
STMN1_HUMAN	P16949	Stathmin	17292	5,76	7	40	74	Sp
NPM_HUMAN	Q8WTW5	Nucleophosmin	32726	4,64	15	30	60	Sp

a Abb, abbreviation.

b AC, accession number.

c MW, molecular weight.

d pI, isoelectric point.

e NVM, number of matched mass values on number of total mass values searched.

f C%, the sequence coverage, which is calculated as the percentage of identified sequence to the complete sequence of the matched protein.

g MS, mascot score.

h CC, culture condition.

**Table 5:** Proteins identified by MALDI-TOF only in ONS-76 Adherent and Sphere cell lines.

*Abb* a	*AC* b	*name*	*MW* c	*p.I.* d	*MVM* e	*C%* f	*MS* g	*CC* h
ARFRP_HUMAN	Q13795	ADP-ribosylation factor-related protein 1	22614	07∶50	9	12	50	Adh/Sp
CH60_HUMAN	P10809	60 kDa heat shock protein, mitochondrial	61187	5,7	10	18	80	Adh/Sp
CHSTE_HUMAN	Q8NCH0	Carbohydrate sulfotransferase 14	43369	9,55	5	25	62	Adh/Sp
FUBP1_HUMAN	Q96AE4	Far upstream element-binding protein 1	67690	7,18	7	16	63	Adh/Sp
GSHB_HUMAN	P48637	Glutathione synthetase	52523	5,67	15	39	178	Adh/Sp
H2B1B_HUMAN	P33778	Histone H2B type 1-B	13942	10,31	5	46	80	Adh/Sp
H2B1C_HUMAN	P62807	Histone H2B type 1-C/E/F/G/I	13898	10,31	9	43	84	Adh/Sp
H2B1D_HUMAN	P58876	Histone H2B type 1-D	13928	10,31	9	43	84	Adh/Sp
H2B1H_HUMAN	Q93079	Histone H2B type 1-H	13884	10,31	9	43	84	Adh/Sp
H2B1J_HUMAN	P06899	Histone H2B type 1-J	13896	10,31	5	46	80	Adh/Sp
H2B1K_HUMAN	O60814	Histone H2B type 1-K	13882	10,31	6	54	80	Adh/Sp
H2B1L_HUMAN	Q99880	Histone H2B type 1-L	13944	10,31	9	43	84	Adh/Sp
H2B1M_HUMAN	Q99879	Histone H2B type 1-M	13981	10,31	9	43	84	Adh/Sp
H2B1N_HUMAN	Q99877	Histone H2B type 1-N	13914	10,31	9	43	84	Adh/Sp
H2B1O_HUMAN	P23527	Histone H2B type 1-O	13898	10,31	5	46	80	Adh/Sp
H2B2E_HUMAN	Q16778	Histone H2B type 2-E	13912	10,31	5	46	80	Adh/Sp
HDGF_HUMAN	P51858	Hepatoma-derived growth factor (HDGF)	26788	4,7	6	22	56	Adh/Sp
HMGB1_HUMAN	P09429	High mobility group protein B1	25049	5,62	7	38	72	Adh/Sp
HNRH1_HUMAN	P31943	Heterogeneous nuclear ribonucleoprotein H	49484	5,89	13	42	150	Adh/Sp
HSP71_HUMAN	P08107	Heat shock 70 kDa protein 1A/1B	70294	5,48	8	18	82	Adh/Sp
ICAL_HUMAN	P20810	Calpastatin	76925	4,98	9	19	79	Adh/Sp
K1328_HUMAN	Q86T90	Uncharacterized protein KIAA1328	66187	8,36	8	17	61	Adh/Sp
KCY_HUMAN	P30085	UMP-CMP kinase	22436	5,44	6	34	70	Adh/Sp
LMNA_HUMAN	P02545	Prelamin-A/C	74380	6,57	10	19	78	Adh/Sp
NPM_HUMAN	P06748	Nucleophosmin	32,575	4,64	11	17	52	Adh/Sp
OVCA2_HUMAN	Q8WZ82	Ovarian cancer-associated gene 2 protein	24,417	6,43	12	18	54	Adh/Sp
PDLI1_HUMAN	O00151	PDZ and LIM domain protein 1	36505	6,56	13	39	148	Adh/Sp
PHB_HUMAN	P35232	Prohibitin	29843	5,57	12	61	174	Adh/Sp
PON1_HUMAN	P27169	Serum paraoxonase/arylesterase 1	39877	5,08	7	24	66	Adh/Sp
PPIA_HUMAN	Q9UC61	Peptidyl-prolyl cis-trans isomerase A	18229	7,68	9	67	104	Adh/Sp
SFPQ_HUMAN	P23246	Splicing factor, proline- and glutamine-rich	76216	9,45	10	22	85	Adh/Sp
SODC_HUMAN	Q6NR85	Superoxide dismutase [Cu-Zn]	16154	5,7	5	38	67	Adh/Sp

a Abb, abbreviation.

b AC, accession number.

c MW, molecular weight.

d pI, isoelectric point.

e NVM, number of matched mass values on number of total mass values searched.

f C%, the sequence coverage, which is calculated as the percentage of identified sequence to the complete sequence of the matched protein.

g MS, mascot score.

h CC, culture condition.

Calpastatin (ICAL) plays a role in neural stem cell self-renewal and differentiation. It is a protein of the calcium-dependent cysteine protease system, binds to a calmodulin- binding site (CALL4) and is also involved in the proteolysis of the amyloid precursor protein.

Poc1 is a stable basal body constituent that localizes to the basal body cartwheel, the site of new basal body assembly, and microtubule cylinder walls. Poc1 is required for stability of basal bodies, ciliary-based motility, and correct cilia formation [15]. Interestingly, calpain and calpastatin activities are modulated during neural differentiation of rat pheochromocytoma (PC12) cells. Altered expression levels for both calpain and calpastatin proteins were also described during human neuroblastoma cell differentiation to Schwann and neuronal cells. Nevertheless, the potential function of calpain during neural differentiation is still poorly understood and requires further investigation [16].

### Immunostaining of Tissue Micro Arrays (TMA)

TMA sections were inspected after scanning with Panoramic Desk (3DHistech). A comparison on the same virtual slide of cells grown in adherence and as spheres enabled confirmation of the proteomic data on the expression of NPM that was negative in adherent UW228 cells but strongly expressed in the corresponding spheres.

Expression of CD44 and CD133 was in accordance with cytofluorimetric data but membrane staining was generally weaker in comparison to cytoplasmic or nuclear antigens.

**Figure 6:**
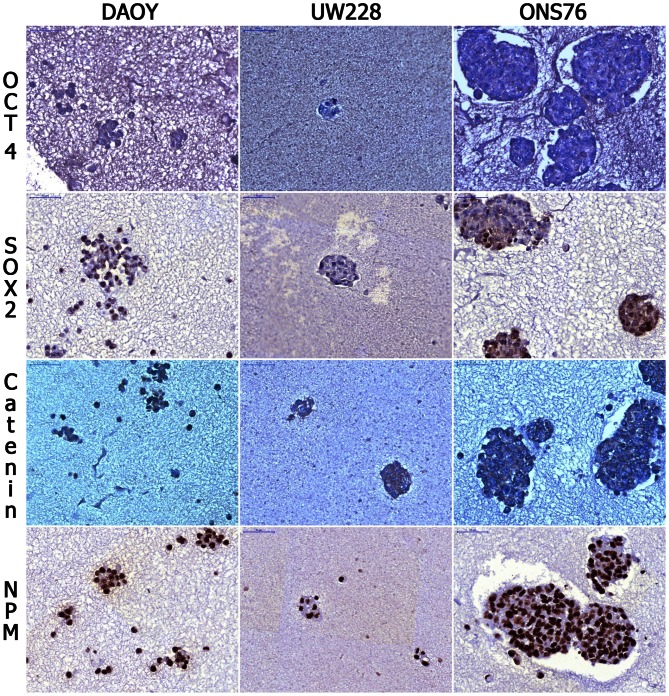
Immunostaining of TMA. Staining for Oct-4, Sox-2, beta Catenin and Nucleophosim on DAOY, ONS76 and UW228 cell lines grown as spheres are shown at 40x magnification. UW228 (second column) show smaller spheres and less intense staining for Oct-4 and Sox-2.

In Figure 6, the microscopical appearance of spheres stained for Oct-4, Sox-2, beta Catenin and NPM are reproduced at 40x magnification.

### Network Generation

**Figure 7:**
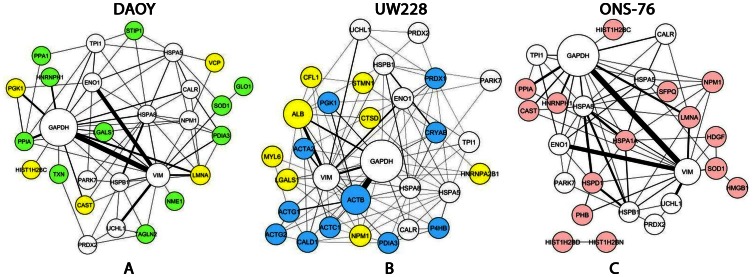
Stem cell Network. The networks identified connect all proteins (nodes) already studied in Stem Cell in the literature. The node size is proportional to the number of papers where the proteins have been studied, while the edge width is proportional to the document number where two proteins have been described together. In the network representation, the group of nodes, white colored, identify the proteins commonly detected in Adh/Sp of the cells analyzed: DAOY (**A**), UW228 (**B**) and ONS-76 (**C**). The proteins overexpressed only in the Sp of each cell types are yellow colored. The specific cell type proteins identified in both Adh/Sp are green colored for DAOY, blue for UW228 and pink for ONS-76.

The networks identify all proteins (nodes) already studied in stem cell literature (Fig. 7). The node size is proportional to the number of papers where the proteins have been studied, while the edge width corresponds to the document number where two proteins have been described together. Usually the biggest node corresponds to the most known protein because either it is the central node of a certain pathway or because it represents a positive control. In the network representation, the group of nodes, white colored, identify the proteins commonly detected in adherent vs. spheres of the cells analyzed: DAOY, UW228 and ONS-76. The proteins overexpressed only in the spheres of each cell types are yellow colored and the others are differently colored in each cell type. There no specific spheres proteins over expression in the ONS-76.

Furthermore only the proteins already identified in the Stem Cell were automatically selected by Protein Quest (PQ). So the proteins network represent a sub-group of the one experimentally analyzed. These aspect support their specific cell type expression and possibly help to further characterized their stem cell features.

The Bingo Gene Ontology was performed to easily identify all Biological Processes (BP) described by adherent vs. spheres proteins. The results are summarized in the Table 6. All cell type have different Biological Processes (p-value <10^−07^–10^−04^) in common characterizing few stem cell features: response to biotic stimulus, negative regulation of programmed cell death, oxygen and reactive oxygen species metabolic and regulation of protein kinase activity. These BP are represented by both common and specific cell type genes.

**Table 6:** Biological Process Analysis MB cells.

			*DAOY cells*	
*p-value*	*n of genes*	*%*	*biological process*	*gene symbol*
1,86E-05	7	25	response to biotic stimulus	HIST1H2BC|VCP|HSPB1|PRDX2|HSPA5|HSPA8|ENO1
4,59E-05	3	11	ER-nucleus signaling pathway	VCP|LMNA|HSPA5
7,86E-05	6	21	negative regulation of programmed cell death	NPM1|HSPB1|GLO1|PRDX2|HSPA5|SOD1
1,72E-04	4	14	nucleocytoplasmic transport	PDIA3|NPM1|LMNA|CALR
1,76E-04	4	14	nuclear transport	PDIA3|NPM1|LMNA|CALR
1,76E-04	4	14	glucose metabolic process	TPI1|PGK1|GAPDH|ENO1
2,70E-04	3	11	oxygen and reactive oxygen species metabolic process	PRDX2|SOD1|PARK7
3,11E-04	5	18	generation of precursor metabolites and energy	TPI1|TXN|PGK1|GAPDH|ENO1
4,98E-04	8	29	regulation of signaling pathway	LGALS1|NPM1|UCHL1|PRDX2|HSPA5|SOD1|CALR|PARK7
5,75E-04	7	25	homeostatic process	PDIA3|TXN|NPM1|PRDX2|SOD1|CALR|PARK7
6,69E-04	5	18	regulation of protein kinase activity	NPM1|UCHL1|PRDX2|HSPA5|SOD1
			**UW228 cells**	
***p-value***	***n of genes***	***%***	***biological process***	***gene symbol***
2,73E-06	8	27	response to biotic stimulus	ACTA2|CFL1|HSPB1|PRDX2|STMN1|HSPA5|HSPA8|ENO1
4,28E-06	4	13	glucose catabolic process	TPI1|PGK1|GAPDH|ENO1
9,73E-06	4	13	oxygen and reactive oxygen species metabolic process	CRYAB|PRDX2|PRDX1|PARK7
1,08E-05	7	23	negative regulation of programmed cell death	CRYAB|ALB|CFL1|NPM1|HSPB1|PRDX2|HSPA5
3,07E-05	7	23	cytoskeleton organization	ACTC1|CRYAB|CALD1|CFL1|NPM1|STMN1|CALR
1,56E-04	5	17	actin filament-based process	MYL6|ACTC1|CALD1|CFL1|CALR
8,28E-04	8	27	regulation of signaling pathway	LGALS1|NPM1|UCHL1|PRDX2|HSPA5|CALR|PRDX1|PARK7
9,00E-04	7	23	homeostatic process	P4HB|PDIA3|NPM1|PRDX2|CALR|PRDX1|PARK7
7,02E-03	4	13	regulation of protein kinase activity	NPM1|UCHL1|PRDX2|HSPA5
			**ONS-76 cells**	
***p-value***	***n of genes***	***%***	***biological process***	***gene symbol***
8,12E-07	8	29	response to biotic stimulus	HIST1H2BC|HSPB1|HSPA1A|PRDX2|HSPD1|HSPA5|HSPA8|ENO1
3,82E-06	7	25	negative regulation of programmed cell death	NPM1|HSPB1|HSPA1A|PRDX2|HSPD1|HSPA5|SOD1
1,76E-05	6	21	cellular macromolecular complex assembly	HIST1H2BC|HIST1H2BD|HIST1H2BN|NPM1|HSPD1|CALR
2,15E-05	4	14	protein-DNA complex assembly	HIST1H2BC|HIST1H2BD|HIST1H2BN|NPM1
3,62E-05	4	14	DNA recombination	HMGB1|PPIA|SFPQ|HSPD1
2,16E-04	3	11	oxygen and reactive oxygen species metabolic process	PRDX2|SOD1|PARK7
4,67E-04	5	18	regulation of protein kinase activity	NPM1|UCHL1|PRDX2|HSPA5|SOD1
2,24E-03	3	11	nucleocytoplasmic transport	NPM1|LMNA|CALR
2,28E-03	3	11	nuclear transport	NPM1|LMNA|CALR
2,28E-03	3	11	glucose metabolic process	TPI1|GAPDH|ENO1
1,65E-03	7	25	regulation of signaling pathway	NPM1|UCHL1|PRDX2|HSPA5|SOD1|CALR|PARK7

For example negative regulation of programmed cell death are commonly represented by NPM1, HSPB1, PRDX2, HSPA5 and specifically by GLO1, SOD1 for DAOY, CRYAB ALB, CFL1 for UW228 and HSPA1A, HSPD1, SOD1 for ONS-76 cells [17], [18].

The specific cell type BP have been also identified for DAOY such as: ER-nucleus signaling pathway p-value (p-value <10^−05^) nucleocytoplasmic transport (p-value<E-04) and generation of precursor metabolites and energy (p-value<E-04). Notably these BP are also enriched by spheres over expressed protein VCP, LMNA and PGK1.

For the UW228 the specific BP are the protein cytoskeleton organization (p-value <10^−05^) and actin filament-based process (p-value <10^−04^). Even in this case some spheres protein are described in these processes: NMP1, CFL1, MYL6 and STMN1.

Finally also the specific BP of the ONS-76 have been identified and are related to cellular macromolecular complex assembly, protein-DNA complex assembly and DNA recombination (p-value <10^−05^).

To further characterized the network, the Genemania analysis was performed.

Genemania generated network represented by official symbol genes including other genes, grey colored, added at the network because strictly connected to genes analyzed.

The network generated include co-expression, co-localization, genetic interaction, physical interaction, predicted and shared protein domain [19].

The networks of each cell type were analyzed. The most connected nodes are represented by co-expression and co-localization data (data not shown). However, in order to define other information compare to the previous analysis, only the protein-protein interactions (physical interactions) were evaluated.

**Figure 8:**
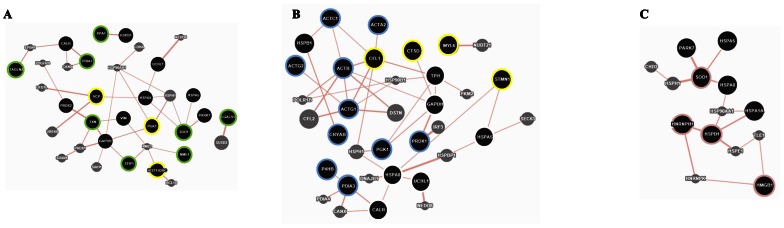
The physical interactions network. Protein-protein interaction are identify by pink edges. The black nodes, white edged represent the physical interactions of the common proteins differentially expressed in all DAOY, UW228 and ONS-76 adherent and sphere cell lines, while Sp overexpressed proteins are yellow edged and specific cell type DAOY(**A**), UW228 (**B**) and ONS-76 (**C**) Sp/Ads are green, blue and pink edged respectively. Grey proteins represented new interactions identified in Genemania proteomic databases. The edge width correspond to the weight of interaction.

In the DAOY, UW228 and ONS-76 the data of physical interactions among proteins represent respectively the 11.8%, 9.64% and 6.86% of networks generated. All the interactions have been represented in the Fig. 8.

The physical interactions were defined for common proteins differentially expressed in adherent and sphere cell lines analyzed: 9/11 of common proteins interact, comprising CARL, GAPDH, HPA5, HSPA8, PARK7, PRDX2, UCHL1 and VIM for DAOY, 7/11 including CARL, GAPDH, HSPA5, HSPA8, TPI1, HSPB1 and UCHL1 for UW228 and 3/11 corresponding to PARK7, HSPA5, HSPA8 for ONS-76. Few interactions were also identify for spheres over expressed protein: VCP, HIST1, H2BC and PGK1 in the DAOY and CTSD, CFL1, STMN1and MYL6 in UW228.

It was also possible to identify new type of interactions, represented by the proteins retrieved in the proteomic database, grey colored.

The edge weight measures relative contribute of information in the network. The physical interactions were evaluated using the default measure weighting method.

## Discussion

Medulloblastoma (MB), the most frequent malignant brain tumor in the pediatric age, has been studied in every aspect from pathological to molecular, in order to guide multimodal therapy to avoid as far as possible overtreatment and therapy-related permanent side effects on developing brain. The propensity of MB to leptopmeningel spreading is an important clinicopathological feature and possibly related to early appearance of invasive clone(s) [20]. Morphological and molecular heterogeneity of this malignant childhood brain tumor has been deeply investigated, and activation of gene and protein expression may allow molecular sub-grouping and dissection of the signaling pathways involved in tumor growth and progression [2], [3]. The presence of a subpopulation expressing stem cells markers (for example, CD133 and Nestin) with the ability to differentiate into more mature phenotype and manifesting resistance to cytotoxic drugs due to a high drug efflux capacity [21] is the basis of the concept of Cancer Stem Cells (CSC) in almost every aggressive tumor, especially in neuro-oncology [22].

Although the CSC model has raised a large interest as an innovative approach to find new therapeutic approaches for reducing tumor resistance, the actual existence of CSC is only putative, and mainly sustained by phenotypic data and the capacity to differentiate.

The presence of markers shared with stem cells (SC) of different origins could be just a recapitulation of embryogenesis, as opposed to a true functional similarity to stemness potentiality.

Besides the issue of CSC, the comparison of protein expression in different growth conditions may give interesting insights related to growth and dissemination of tumor cells in the natural history of the disease, and possibly new data related to resistance to treatment and recurrence.

One interesting observation is the fact that, although the same growth conditions were sufficient to obtain medullospheres from the three cell lines a striking difference of size was note for ONS76. This phenomenon is observed also in neural SC form adult brain and is explained to a different sensitivity to growth factors depending on the site of origin in CNS and the concentration of cytochines in the media [23]. For CSC this aspect may be even more evident with the impossibility of obtaining sphere formation from some cell lines but not from other from tumors of the same histotype [24].

The comparison of three established cell lines of MB cultured in adherence, or as medullospheres, was aimed at unravelling the influence of growth conditions on the modulation of protein expression.

*In vitro* invasion capability analysis empathized MB cell lines heterogeneity. ONS-76, already described as the most immature cell line, showed, in addition, the most great transwell migration capability. DAOY migrated less than ONS-76 but much more than UW228, known to be less invasive [9], [10]. On the other hand, these specificities seem to be reduced in MBS since all spheres showed a very little invasion capability. If this couldn’t seem intuitive, in basis of the concept of CSC in aggressive tumors [21], we can suppose that MBS are more resistance to treatments but less pressured to invasion.

The DAOY proteomic profile is extensively described in Peyrl et al. (2003) [25] with a list of more than 200 identified proteins; however, when the DAOY cell line grows as spheres, eight or nine new proteins are identified by mass spectrometry that have not previously been described. Conversely, since no detailed data were previously described for UW228 and ONS-76 cells, our report, as far as we know, is the first proteomic characterization of these lines.

In contradiction to what was expected, few modifications in the proteomics asset were found, and were limited to the DAOY and UW228 spheres.

No new proteins were expressed by ONS-76 cells, although quantitative modifications of expression were detected. Of particular note is the protein NPM: already present in DAOY and ONS-76 under adherence conditions, it is expressed as a new protein in UW228 spheres, and is the only protein common in all three MBS. NPM has been extensively studied in human lymphomas [26], leukemia [13] and colon cancer [27] but not in MB. The expression of this protein in basal conditions for DAOY and ONS-76 and the appearance in UW228 spheres may be correlated to the ability of MB to survive in less favorable growth conditions.

Conversely, in DAOY and UW228, new proteins, not present in adherence conditions, appeared, but without any shared protein between the two lines.

Among the newly expressed proteins, Stathmin was identified, although it was exclusively expressed by UW228 spheres. This protein is described as a marker of aggressiveness.

High stathmin expression has been correlated with tumor dissemination, is an important prognostic factor of medulloblastoma, and may serve as a useful marker for more intensive adjuvant therapy [28].

GeneOntology analysis showed that all cell types have different Biological Processes (BP) (p-value <10^−7^–10^−4^) in common, some characterizing stem cell features: response to biotic stimulus, negative regulation of programmed cell death, oxygen and reactive oxygen species, metabolic and regulation of protein kinase activity [18], [19]. These analysis of experimental data was also performed with Genemania, that integrates different sources of data such as literature and other publicly available biological datasets to easily identify the most related genes or proteins in each specific cell type. The protein-protein interactions were characterized for each network. Even more additional interactions were retrieved by the connected genes proteins added from Genemania dataset. These data could offer new perspective of study.

Considering all these results, it is remarkable that ONS-76 tumor cells display a more immature or primitive profile, expressing at least some SC markers even when cultured in adherence conditions, and showing only minor modifications induced by the conditioning to spheres. Ultrastructural analysis confirmed that ONS-76 MB cell line feature the undifferentiated phenotype, as evident in MBS. This aspect is in contrast with the evident proteomic changes induced in DAOY and UW228 cell lines when grown as spheres, which show undifferentiated aspects in both cell lines, i.e. the high nucleus:cytoplasmic ratio rather than minimal aspects of differentiation, such as a rudimentary RER in a fairly well represented cytoplasm, as seen in MB cell lines.

Immunohistochemistry confirmed stemness enrichment of spheres, showing expression of Sox-2, a marker of neural stem cells, and increased expression of NPM, a resistance marker.

A recent report by Wu et al. (2012) [20], demonstrating tumor heterogeneity as a relevant mechanism of MB for metastatic spread to leptomeningeal spaces of aggressive clones that have lost markers of the primitive tumor, may be interpreted as being in contrast to the CSC model, with the explanation of clonal evolution of aggressive and more “primitive” tumor cells that may express a stem-like phenotype [29].

Conversely, an impressive simplification of the proteomic asset of MB was reported in a relapsing MB after radiotherapy with the expression of some new proteins [14].

While sphere formation may not be resolving as far as the issue of CSC, it may however be important for dissection of the adaptive modifications of tumor cells from primary or stabilized cell lines in an environmental context less permissive than adherence culture and with limited oxygen [30] and nutrient gradient availability. A detailed knowledge of such adaptive capabilities may give possible suggestions for new treatment approaches for complete eradication of tumor cells.

## Materials and Methods

### Chemicals and Reagents

MEM/EBSS, fetal bovine serum, sodium pyruvate, non-essential amino acids (NEAA), 2 mL-Glutamine, streptomycin and penicillin, DMEM/F12 Glutamine, RPMI, EUROMED CSC Neuronal medium, streptomycin and penicillin were obtained from EuroClone, Pero, Italy. Anti-CD44 and anti-Stathmin were purchased from Cell Signaling (Danvers, MA, USA). Anti-CD133, anti-Oct4, anti-Nanog, anti-Nestin, anti-Sox-2 mouse and goat polyclonal secondary anti-rabbit IgG H&L FITC-conjugated were purchased all from AbCam (Cambridge, UK). Transwell membrane was purchased from NeuroProbe Inc, (Gaithersburg, MD) and Matrigel from BD Bioscience (Bedford, MA). Anti- β-catenin, anti-Nucleophosmin (NPM) were purchased from Santa Cruz (Heidelberg, Germany). Fixation/Permeabilization solution was purchased from (BD). Ki67 was purchased from Ventana, Martinengo, Italy. DC Protein assay kit, acrylamide, agarose, readymade immobilized pH gradient (IPG) strip (7-cm IPG strips pH 3–10NL) were purchased from Bio-Rad (Hercules, CA, USA). Ampholine pH 3.5–10 was obtained from GE Healthcare (MI, ITALY). May-Grunwald Giemsa solution, protease inhibitors, ammonium persulfate (APS), bromophenol blue, glycerol, N,N,N’,N’-tetramethylethylene-diamine (TEMED), sodium dodecyl sulfate (SDS), TRIZMA, urea, 3-[(3-cholamidopropyl) dimethylammonio]-1-propanesulphonate (CHAPS), dithiothreitol (DTT), iodoacetamide were purchased from Sigma-Aldrich (St. Louis, MO, USA).

### Culture of MB Cell Lines and MBS Generation

DAOY cells line was purchased from ATCC (United Kingdom U.K.), UW228 [31] and ONS-76 [9] cell lines were kindly provided by Dr. Charles G. Eberhart (John Hopkins University, Baltimora, MD) with the agreement of Dr. Mike Bobola (University of Washington, Seattle, WA).

MB cell lines DAOY, UW228 and ONS-76 were cultured at 37°C, in 5% CO_2_. DAOY (3×10^4^/ml) was cultured in MEM/EBSS supplemented with 10% heat-activated fetal bovine serum, sodium pyruvate, non-essential amino acids (NEAA), 2 mL-Glutamine, 100 g/mL streptomycin and 100 U/mL penicillin; UW228 (5×10^4^/ml) was cultured in DMEM/F12 supplemented with 10% heat-activated fetal bovine serum, 2 mL-Glutamine, 100 g/mL streptomycin and 100 U/mL penicillin and finally ONS-76 was cultured (3×10^4^/ml) in RPMI supplemented with 10% heat-activated fetal bovine serum, NEAA, 2 mL-Glutammina, 100 g/mL streptomycin and 100 U/mL penicillin. In order to produce MBS, DAOY, UW228 and ONS-76 were grown at confluence in adhesive condition, trypsinized, pelleted and plated (6×10^4^/ml) in ultra-low attachment T25 Flasks (Corning Inc., NY, USA) for a further 7 days in serum-free EUROMED CSC Neuronal medium. After the first passage (P1) MBS were spun down at 1400 rpm (368 g), harvested and resuspended as a single cells suspension in the same serum-free medium at the same concentration in order to obtain subsequent passages. The spheres were identified as spherical ovoid aggregates with smooth outlines of more then 20 cells under microscope observation. For each passage, sphere suspensions from all cell lines (50 µl) were collected and counted in 96-well plates by inverted microscope (Olympus CKX41).

### Morphological and Electron Microscopy of MB and MBS Cell Lines

MB cells (DAOY, UW228 and ONS-76) and their corresponding MBS morphology were investigated with phase contrast by inverted microscopy and photographed with 40X magnification with a digital camera (Leica DFC345 FX). Dimension analysis of spheres was determined using the Panoramic Viewer (3D Histech).

For ultrastructural evaluation all MB cells and MBS were fixed in 2.5% glutaraldehyde in 0.1 M sodium cacodylate-buffer pH 7.3 immediately following *in vitro* culture. Pelleted cells were post-fixed in osmium tetroxide in the same buffer, dehydrated in ethanol and embedded in Araldite. Thin sections stained with uranyl acetate and lead citrate were studied using a Philips 400 T transmission electron microscope.

### Flow Cytometry

Three independent determinations were evaluated to determine MB cells and MBS phenotype and to calculate the average relative positivity and standard deviations for each cell line. MB cells and MBS were incubated with primary antibodies: mouse monoclonal anti-CD44 and rabbit polyclonal anti-CD133 for 30 minutes in FACS Flow (BD, Mi, Italy). Cells and spheres were subsequently incubated with goat polyclonal secondary anti-rabbit IgG H&L FITC-conjugated antibody for 30 minutes in FACS Flow. Alternatively, MB cells and MBS were fixed and permeabilized with Fixation/Permeabilization solution and incubated with rabbit polyclonal anti-Oct4, rabbit polyclonal anti-Nanog, mouse monoclonal anti-Nestin and rabbit polyclonal anti- β-catenin for 30 minutes in Perm/Wash™ buffer. Cells and spheres were subsequently incubated with goat polyclonal secondary antibody to mouse IgG H&L PE-conjugated or goat polyclonal secondary antibody to rabbit IgG H&L FITC-conjugated for 30 minutes in Perm/Wash™ buffer. For each determination at least 10,000 cells were analyzed on a Dako CyAn™-ADP. Summit® software was used for evaluation of results, including dot-plot and percentage of positive cells. The significance of differences between experimental conditions in each determination was determined using the two-tailed Student’s t test. Probability values less than 0.01 were considered significant (*p<0,01).

### In vitro Invasion Assay

To assess cell migration ability in vitro, MB cells, DAOY, UW228 and ONS-76 (30,000 cells in serum-free medium), and their corresponding MBS (30,000 cells in serum-free EUROMED CSC Neuronal medium) were placed in the top chamber of a transwell migration Boyden chamber. The lower chamber was filled with 35 µl of serum (10%) containing medium. The transwell insert contained an 8-µm-pore-sized membrane with a thin layer of Matrigel. After incubation at 37°C for 24 h, cells that had not migrated to the lower chamber were removed from the upper surface of the transwell membrane with a cotton swab. Migrating cells on the lower membrane surface were fixed, stained with a May-Grunwald Giemsa solution and finally, stained cells were counted under a light microscope at 10X magnification. Two independent invasion assays were performed in duplicate. For statistical analysis data were analyzed using unpaired, two-tailed t test and the Graphpad software package for Windows (PRISM5). p<0.05 was considered to be statistically significant.

### Tissue Array and Immunostaining

For light microscopy, cells cultured in adherence and as spheres were inglobated in fibrin mesh according to the cell-block procedure [32] with gentle shaking during the cloth formation in order to obtain a uniform distribution of cells or spheres throughout the inclusion. Cell-blocks were subsequently fixed overnight in a formaldehyde-free fixative as previous described [33]. After routine processing and inclusion in paraffin, pilot sections were cut and stained with Hematoxylin and Eosin.

From paraffin inclusions, tissue micro arrays (TMA) were constructed with TMA-Master (3DHistech, Euroclone, Italy) with 1.5 cores punched out from paraffin-embedded cells and spheres. Tissue sample tumors, such as medulloblastoma, and normal human tissue were also inserted in the TMA as internal controls for immunostaining.

Immunostaining was performed with Sox-2, Stathmin, β-catenin, Nestin, NPM, CD133, OCT4, Nanog, CD44 and Ki67 at the manufacturer’s suggested dilutions (or higher if necessary to avoid background staining). Antigen retrieval was induced by microwave treatment in EDTA buffer pH 8.5 for 5 minutes. Thermo Quanto (Bio-Optica, Milan, Italy) revealing kit with diaminobenzidine as chromogen was used according to the manufacturer’s instructions.

### Two-dimensional Gel Electrophoresis (2-DE)

2-DE was performed using ready-made immobilized pH gradient (IPG) strips (7-cm IPG strips, pH 3–10 NL). Each sample (250 µg of protein for preparative gels) was applied onto an IPG gel by in-gel rehydration for 20 h, adding DTT 1% w/v, final concentration and Ampholine pH 3.5–10, 2% v/v, final concentration.

Isoelectric focusing was carried out in a Protean IEF cell apparatus (Bio-Rad, Segrate, Italy). Briefly, focusing for 7-cm IPG strips was started at 250 V, and the voltage was progressively increased to 4000 V until a maximum of 25000 V/h was reached. Focusing was performed at 18°C with a limit of 50 mA per strip. Subsequently, IPG strips were equilibrated by continuous shaking for 15 min in equilibration buffer 1 (6 M urea, 2% w/v SDS, 0.05 M Tris–HCl pH 8.8, 20% v/v glycerol, 1% w/v DTT) and for 12 min in equilibration buffer 2 (6 M urea, 2% w/v SDS, 0.05 M Tris–HCl pH 8.8, 20% v/v glycerol, 2.5% w/v iodoacetamide). For the second dimension, 7 cm 10% acrylamide gels were run on the Mini Protean system (Bio-Rad). Electrophoresis was performed for 30 min at 50 V and was then continued at 100 V until the Bromophenol blue front reached the lower limit of the gel. Gels were stained with colloidal Coomassie (18% v/v ethanol, 15% w/v ammonium sulfate, 2% v/v phosphoric acid, 0.2% w/v Coomassie G-250) for 48 h and destained with water.

2-DE image analysis was performed using PD-Quest software (version 7.2, Bio-Rad, Hercules, CA) according to the manufacturer’s instructions. Normalization of each individual spot was performed according to the total quantity of the valid spots in each gel, after subtraction of the background values. The spot volume was used as the analysis parameter to quantify protein expression.

### MALDI-TOF: Protein Identification by Mass Spectrometry and Database Search

Coomassie G-stained spots were excised from 2-DE preparative gels; destaining and in-gel enzymatic digestions were performed as previously described [34]. Briefly each spot was destained with 100 ml of 50% v/v acetonitrile in 5 mM ammonium bicarbonate and dried with 100 ml of acetonitrile. Each dried gel piece was rehydrated for 40 min at 4°C in 5 µl of a digestion buffer containing 5 mM ammonium bicarbonate, and 10 ng/ml of trypsin. Digestion was allowed to proceed overnight at 37°C and the peptide mixtures were stored at 4°C until assayed. All digests were analyzed by MALDI-TOF (TofSpec SE, MicroMass) equipped with a delayed extraction unit. Peptide solutions were prepared with equal volumes of saturated a-cyano-4-hydroxycinnamic acid solution in 40% v/v acetonitrile-0.1% v/v trifluoroacetic acid. The MALDI-TOF was calibrated with a mix of PEG (PEG 1000, 2000 and 3000 with the ratio 1∶2∶2) and mass spectra were acquired in the positive-ion mode. Peak lists were generated with ProteinLynx Data Preparation (ProteinLynx Global Server 2.2.5) using the following parameters: external calibration with lock mass using mass 2465.1989 Da of ACTH, background subtract type adaptive combining all scans, performing de-isotoping with a threshold of 1%. The 25 most intense masses were used for database searches against the SWISSPROT database (release 2012-09 of 03-Oct-12) using the free search program MASCOT 2.4.01 (http://www.matrixscience.com). Search settings allowed one missed cleavage with the trypsin enzyme selected, carbamidomethylation of cysteine as a fixed modification, oxidation of methionine as a potentially variable modification, a peptide tolerance of 100 ppm, taxon- human.

### Network Generation

The proteins identified in the three cell types were further analyzed by means of a web platform tool ProteinQuest (PQ) (http://www.proteinquest.com). PQ automatically retrieved all relevant biological information from PubMed abstracts and captions from free full-text articles, US patents and Clinical Trials.

Image captions were extracted using the BFO Java library (http://bfo.com) on the PDF version of the scientific articles [35].

The ProteinQuest (PQ) database contains documents already tagged with biomedical dictionaries and ontologies such as proteins, drugs, cells, miRNA, diseases, bioprocess, clinical and biological techniques, etc. Each term assignment also takes into account the entire correspondent alias, so as to solve any possible disambiguation.

The biological relationship among terms identified into results can be explored by analyzing the co-occurrence of just a couple of terms.

Networks can be a representation of any relationship among terms; using PQ it is possible to generate a network from biological literature data connected by different articles.

Here the protein networks were generated by selecting the genes that are differentially expressed in the experiments described above, already associated with the stem cells in the literature. Networks connecting all proteins from analyzing stem cells described in the literature were visualized using Cytoscape [36], a popular software platform for network analysis, and further characterized by Bingo plugins [37] for Gene Ontology Analysis.

Finally the Genemania plugin [19] was used to easily predict gene functions of each cell type.
